# Original Ideas—Inlays

**Published:** 1891-08

**Authors:** 


### Editorial.

### ORIGINAL IDEAS—INLAYS.

THE value of having a place of record for new ideas was dwelt upon in a previous article, and the importance of this increases with every added month's experience. Ideas apparently of little value at the time of introduction have grown to serious proportions, and some of positive worth have drifted, by the influence of opposing interests, into oblivion.

It is within the cognizance of all that the apparently trivial operation of placing a band on a tooth has led up to crowns and from crowns to bridge-work with all that this implies. Had those who made this work in the experimental stages kept models and placed them on record, the litigation, so familiar, would have been impossible.

The younger generation of dentists have but little conception of the struggle waged by the profession against the encroachments of the company that manipulated the "rubber base," and of the years of annoyance, loss, and positive suffering that this occasioned. Had care been taken at the beginning, patenting would probably have been impossible, or at least valueless. It is the knowledge that carelessness reigns supreme in the dental profession in these vital matters which gives the unscrupulous the power and the audacity to patent improvements already in use. They trust to this want of care, and the trust has been often demonstrated not to have been misplaced, that when the case is tried in the courts there will be no evidence to invalidate the patent.

It is not necessary to multiply evidence in this direction, for the profession has experienced to its great sorrow the depths to which some men can descend in moral dregs, and at the same time how utterly valueless have been the efforts put forth on their part to combat it.

The present organization of the profession promises better results, but only in the direction of close attention to original data, upon which the superstructure of the future must be built.

Our attention has been again called to this matter by reading the discussion recently held upon "The Report of the Committee on Dental Art and Invention," W. B. Ames, D.D.S., chairman, and read before the Illinois State Dental Society (*Dental Review*, June 15, 1891). The remarks drifted to an examination of various kinds of inlays, and, as a whole, was of unusual interest. The point, however, that seemed to impress the writer was a lack of information in regard to the origin of certain forms.

It would be difficult to go back to the period when this operation was first introduced as a means to overcome the disfigurement of a filling on labial surfaces, nor is it important, as it does not affect materially the question at issue.

The impetus given to this subject in this country has been largely due to Dr. W. Storer Howe's efforts to improve the older process, and has had much to do with leading the inventive mind, both here and in Germany, to the adoption of various means to render this of far more value than could have been anticipated even a decade in the past.

In the discussion alluded to, glass inlays were particularly dwelt upon, and the various modes best adapted for taking an impression of the cavity were described at length, as also the material of which to form the inlay, gold, porcelain, and glass each having adherents.

The preparation of the matrix in which to form the inlay called for special attention. That made from gold-foil was preferred where gold or glass inlays were to be used.

It is at this point that we feel that special regard should be had to the origin of the process, as it may contain the germ of something of great value in the future.

At the session of the American Dental Association, held at Excelsior Springs, Mo., 1890, Dr. James A. Swasey described his process as follows:

"The inlay that I use in all large cavities . . . I make from gold. My method is to cut a piece of proper size from a ribbon of gold rolled to about one hundred to one hundred and twenty, and then to anneal it. A piece of cork or erasing rubber is then cut to fit into the cavity. The piece of annealed gold is then placed over the cavity, being held with the pliers in the left hand, while the gold is burnished with a large burnisher into the cavity. The cork or erasing rubber is then put in place and held there, and the edges are then burnished down. The overlapping gold should then be trimmed off, the whole annealed again, and placed in the cavity with rubber or cork in the inside. The mouth should then be closed, when the pressure of the rubber will make an adjustment of the gold on the margin of the cavity. The inlay should then be carefully removed and invested. Twenty-carat gold should then be cut into strips and placed in the shell and melted there."

This is sufficient to show that Dr. Swasey claimed originality in the form and process of making the matrix.

In the November (1890) number of the INTERNATIONAL DENTAL JOURNAL is an article by James A. Bruce, D.D.S., of Melbourne, Australia, in which he describes Dr. Herbst's method, a knowledge of which he acquired by personal observation. The plan of Dr. Herbst will be found practically the same as that already given by Dr. Swasey. After describing the preparation of the cavity without undercuts, he says, "Next line the cavity with one layer of No. 60 gold-foil, pressing it into place with india-rubber or some such material, and finally taking a wax impression (the wax being used hard), the gold being withdrawn in the wax. The result is a sharp impression of the cavity, into which plaster and pumice in proportion of two to one, respectively, is run. After this is thoroughly set, boil out the wax, leaving your model with the gold coating." Into this matrix glass is melted, the gold remaining "giving a sharper edge to the glass filling" (inlay).

Again, in the JOURNAL for March, 1891, Dr. John Girdwood, Edinburgh, enlarges upon this same topic, after giving Drs. Herbst and Richter the credit of the earlier work in glass inlays and the preparation of the matrix of gold, as described, and then gives some original ideas in regard to the melting of glass.

At the Illinois State Dental Society meeting, already alluded to, Dr. Harlan quotes from an article by Mr. Henri Weiss, published regularly in *Ash's Quarterly Circular*, but credit is given here to *The Journal of the British Dental Association*, of April 15, 1891. This is identically the same as the Herbst process and differs in no essential particular from the Swasey mode. Dr. Harlan was evidently misled into regarding the Weiss process as an original one, which from present information it does not seem to be.

The object of this examination is not to determine priority of discovery, but to place facts on record, leaving to others to determine the origin. It seems to us to lie between Drs. Herbst, Richter, and Swasey, but sufficient data are not at hand to determine the matter.

The merit or demerit of the process is not now in question; but it seems as though a new field in practice had been opened, the extent of which it is impossible to realize, and it therefore becomes our duty to guard well the beginnings, that a patent in the future may be made an impossibility.

